# 孤立性肺转移瘤的诊断与外科治疗（附156例报告）

**DOI:** 10.3779/j.issn.1009-3419.2012.04.06

**Published:** 2012-04-20

**Authors:** 辉 张, 晓峰 陈, 海兵 王, 雷 张, 文勇 周, 明川 赵

**Affiliations:** 200433 上海，同济大学附属上海市肺科医院胸外科 Department of Thoracic Surgery, Shanghai Pulmonary Hospital Affiliated to Tongji University, Shanghai 200433, China

**Keywords:** 肺肿瘤, 肿瘤转移, 手术, 诊断, 预后, Lung neoplasms, Metastasis, Surgery, Diagnosis, Prognosis

## Abstract

**背景与目的:**

近年来手术治疗孤立性肺转移瘤取得了满意的疗效，为提高患者的生存率，本研究对其诊断、外科治疗的适应证、手术方式及影响预后的因素进行分析讨论。

**方法:**

对156例接受手术治疗的孤立性肺转移瘤患者的临床资料进行回顾性分析。

**结果:**

原发肿瘤为癌者134例，肉瘤21例，不明组织类型1例。全组无围手术期死亡，随访153例，随访时间1年-10年。术后5生存率为31.2%，中位生存期为35.8个月。113例行淋巴结系统性清扫，淋巴结转移阴性和阳性患者5年生存率分别为37.3%、12.5%。行肺叶切除术患者5年生存率为38.5%。

**结论:**

手术治疗孤立性肺转移瘤可取得满意的疗效，电视胸腔镜手术是有效的手术方式，有无淋巴结转移和肺叶切除方式是影响预后的重要因素。

## Abstract (en)

**Background and objective:**

In recent years, it has been proven that surgical treatment for solitary pulmonary metastases has achieved satisfactory results. Consequently, the study aims to investigate the diagnosis, indications for surgery, operative techniques, and prognostic factors of the surgical resection for solitary pulmonary metastases, and to improve the survival rate of patients with pulmonary metastases.

**Methods:**

The medical records of 156 patients with surgical procedures at our institution were retrospectively reviewed.

**Results:**

The primary tumors were verified as cancer in 134 cases, sarcoma in 21, and 1 contained unknown tissue. There was no perioperative mortality. A total of 153 patients returned for follow up. Follow-up time was 1 yr to 10 yr. The 5-year survival rates were 31.2%. The median survival time was 35.8 months. Systematic lymph node dissection was performed in 113 patients. The 5-year survival rates were 12.5% for lymph node-positive patients and 37.3% for lymph node–negative patients. The patients who underwent lobectomies had better survival rates, with a 5-year survival rate of 38.5%.

**Conclusion:**

Surgery is recommended for patients with solitary pulmonary metastasis if they fulfill the surgical indications and favorable outcomes can be achieved. VATS can be chosen for the patients. Hilar and mediastinal lymph node involvement and the surgical approach are potentially important prognostic factors.

近年来手术治疗部分选择性肺转移瘤患者取得了满意的疗效^[1-4]^，为进一步提高孤立性肺转移瘤患者的诊疗水平，本研究对1967年9月-2010年9月在同济大学附属上海市肺科医院胸外科治疗的156例孤立性肺转移瘤患者的临床资料进行了回顾性分析，以探讨孤立性肺转移瘤的诊断、外科治疗的适应证、手术方式以及与预后有关的因素。

## 1 材料与方法

### 1.1 临床资料

全组共156例孤立性肺转移瘤患者接受手术治疗(表 1)，男96例，女60例；年龄9岁-75岁，中位年龄54岁。53例(34%)因出现症状就诊而发现，主要症状为胸痛、胸闷、咳嗽、咳痰伴痰中带血、发热等。103例(66%)为原发肿瘤治疗后常规复查过程中发现。21例同时发现原发肿瘤与肺转移瘤，其余患者无病生存期(disease free interval, DFI)最长为215个月，平均37.7个月。原发肿瘤为癌者134例(85.9%)，其中结直肠癌48例，肾癌12例，乳腺癌24例，鼻咽癌8例，肝癌11例，食管癌、子宫颈癌、胃癌、甲状腺癌各3例，贲门癌、绒毛膜上皮癌、胰腺癌、喉癌、黑色素瘤、皮肤癌各2例，腺体癌、精原细胞癌、前列腺癌、阴茎癌、畸胎瘤、恶性胸腺瘤、脊索癌各1例。肉瘤21例(13.5%)，其中成骨肉瘤12例，软骨肉瘤3例，纤维肉瘤、平滑肌肉瘤、滑膜肉瘤各2例。原发肿瘤组织学类型不明1例(0.6%)。

**1:** 孤立性肺转移瘤患者术后生存因素的单因素分析 Univariate analysis of prognosis of the 156 patients with solitary pulmonary metastases resected

Risk factors	*n* (%)	*P*
Age (year)		0.390
≤60	89 (57)	
> 60	67 (43)	
Gender		0.819
Male	96 (62)	
Female	60 (38)	
Location		0.753
Central	71 (46)	
Peripheral	85 (54)	
Tumor diameter (cm)		0.790
≤2	24 (15)	
2.1-3	38 (24)	
3.1-5	47 (30)	
5.1-7	34 (22)	
≥7.1	13 (9)	
N disease		< 0.000, 1
Positive	21 (13)	
Negative	92 (59)	
Unknown	43 (28)	
Surgery^*^		< 0.000, 1
Lobectomy	63 (35)	
Bilobectomy	110 (62)	
Pneumonectomy	5 (3)	
Histological type		0.371
Breast cancer	24 (15)	
Head and neck cancer	13 (8)	
Digestive system cancer	67 (43)	
Urinary system cancer	16 (10)	
Osteosarcoma	21 (14)	
Other	15 (10)	
^*^A total of 178 surgical procedures were performed on 156 patients.		

### 1.2 治疗

全组患者经术前评估均可耐受手术，术前检查未发现肺外转移灶。胸部X线片和计算机断层扫描(computed tomography, CT)检查提示为单侧孤立性单发肿物，其中58例(37.2%)有分叶、边缘毛刺、偏心空洞及胸膜凹陷征的表现；98例(62.8%)为边缘清楚、密度均匀的类圆形肿块。17例肺转移瘤直径 < 1 cm，经随访3个月到半年后发现结节明显增大，遂采取手术治疗。全组共实施手术178次，其中行1次手术136例(87.2%)，行2次手术18例(11.5%)，行3次手术2例(1.3%)。手术方式主要根据肺转移瘤的部位、大小来决定。行不规则肺部分切除术(包括肺楔形切除术)87次(48.9%)，肺段切除术23次(12.9%)，肺叶切除术63次(35.4%)，由于肿瘤较大且位于肺门部行全肺切除5次(2.8%)，其中开胸手术104次(58.4%)，行电视胸腔镜手术(video-assisted thoracic surgery, VATS)74次(41.6%)。113例患者行肺门及纵隔淋巴结系统性清扫，其中全肺切除术和肺叶切除术患者均进行了系统性淋巴结清扫，行不规则肺部分切除术和肺段切除术的患者部分进行了系统性淋巴结清扫。全组患者术后根据相应的病理类型给予辅助性化疗。

### 1.3 统计方法

应用SPSS 18.0软件进行统计分析。生存分析采用*Kaplan-Meier*法，组间比较采用*Log-rank*检验；采用*Cox*比例风险模型对影响预后的因素进行多因素分析。*P* < 0.05为差异有统计学意义。

## 2 结果

### 2.1 疗效与预后

全组无围手术期死亡病例。12例术后出现并发症，其中心率失常8例，呼吸衰竭1例，肝功能不全1例，乳糜胸1例，肺部感染合并心率失常1例，经治疗后均顺利恢复。全组随访153例，随访时间1年-10年，随访率为98.1%，随访形式主要采用电话随访，部分患者采用信访。本次随访中有3例患者失访，18例患者死于其它疾病，失访及死于其它疾病患者的生存期算作截尾数据。本组患者中位生存时间为35.8个月。1年、3年、5年、7年及10年生存率分别为83.3%、46.5%、31.2%、22.8%、15.7%(图 1)。113例患者行肺门及纵隔淋巴结系统性清扫，21例(18.6%)术后病理证实有淋巴结转移。无淋巴结转移患者和有淋巴结转移患者5年生存率分别为37.3%和12.5%。原发肿瘤以头颈部癌、结、直肠癌和软组织肉瘤的预后较好，5年生存率分别为41.2%、37.8%、36.9%。行肺叶切除术患者的预后较好，5年生存率为38.5%；行肺段切除术和楔形切除术患者的5年生存率分别为29.3%和28.4%，与肺叶切除术患者5年生存率相比差异有统计学意义(*P* < 0.05)。行VATS或开胸手术患者的5年生存率分别为33.3%和27.4%，两组相比差异无统计学意义(*χ*^2^=0.590, *P*=0.442)(图 2)。

**1:**
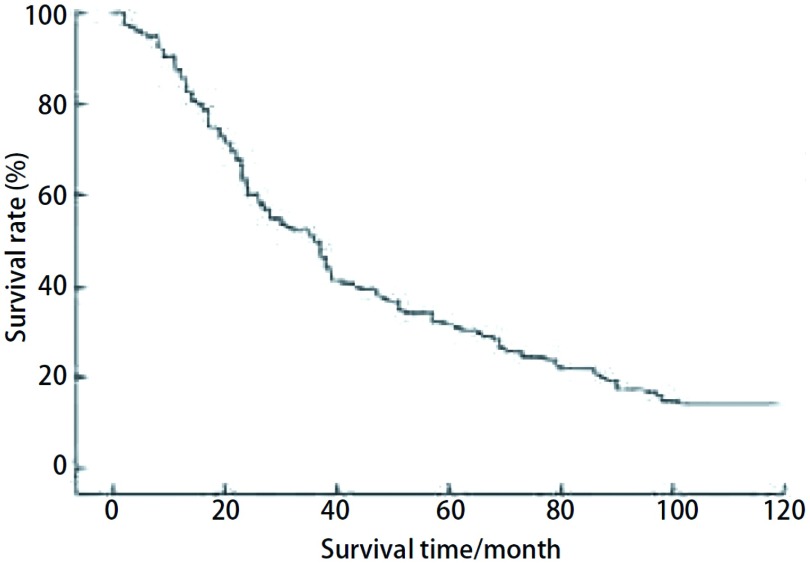
156例孤立性肺转移瘤患者术后生存曲线 Postoperative survival curve of the 156 patients with solitary pulmonary metastases

**2:**
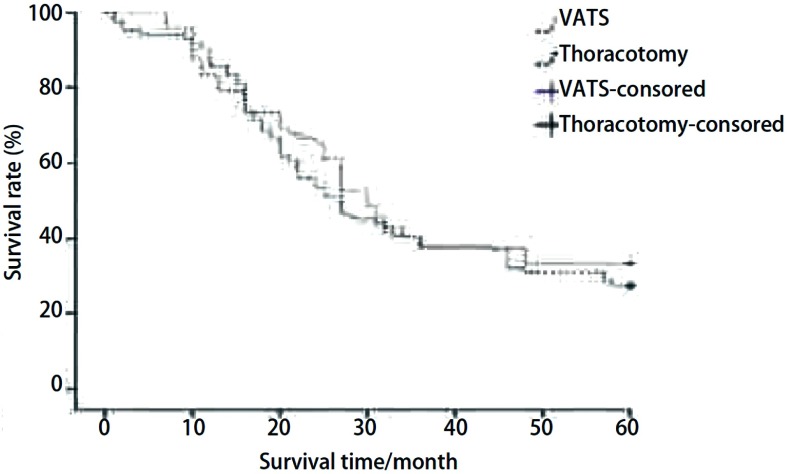
VATS组与开胸组患者术后生存分析（*P*=0.442） Surivival analysis between the VATS group and the thoracotomy group (*P*=0.442). VATS: video-assisted thoracic surgery.

### 2.2 影响预后的相关因素分析

有无淋巴结转移和肺叶切除方式是影响患者术后生存的独立因素。无淋巴结转移、采取肺叶切除术的患者预后较好(表 2)，而年龄、性别、转移瘤大小、转移瘤在肺部的位置、原发肿瘤病理类型和采取VATS或开胸手术与患者的预后无关。

**2:** 孤立性肺转移瘤患者术后生存因素的多因素分析 Multivariate analysis of prognosis of the 156 patients with solitary pulmonary metastases resected

Factor	Coefficient of regression	Standard error	*Wald* value	*P*	Relative risk	95%CI
Lymph node	1.187	0.268	19.57	< 0.000, 1	3.279	1.937-5.548
Surgical approach	1.041	0.176	34.01	< 0.000, 1	2.832	1.996-4.018
CI: confidence interval.						

## 3 讨论

### 3.1 孤立性肺转移瘤的诊断及影像学检查

大多数孤立性肺转移瘤患者早期症状并不明显，在本次研究的全部病例中，53例(34%)因出现胸痛、胸闷、咳嗽等症状而就诊，大多数患者是在原发肿瘤治疗或随访过程中发现。因此关于肺转移瘤的术前诊断主要依据患者是否有恶性肿瘤的病史及影像学检查。孤立性肺转移瘤在胸部X线和胸部CT上主要表现为单发的圆形、类圆形肿块，也可表现为单发的结节影，部分病例还可表现为形状不规则、边缘呈毛刺状的典型原发型肺癌的影像学特点。本次研究有58例具有原发性肺癌的典型影像学特点，经术后病理学诊断确诊为肺转移瘤。对于术前影像学检查提示为小结节的患者，需要进行短期的影像学观察。本次研究中有17例患者术前影像学检查提示为小结节，在随访过程中出现结节增大，遂实施手术，经术后病理学检查证实为肺转移瘤。

有学者报道了高分辨率计算机断层扫描(high-resolution computed tomography, HRCT)及正电子发射断层与计算机断层成像(positron emission computed tomography, PET-CT)对诊断肺转移瘤的灵敏度。Pfannschmidt等^[5]^报道HRCT在扫面厚度分别为3 mm和5 mm时对诊断肺转移瘤的灵敏度分别达到88.8%和83.7%，对诊断1 mm-4 mm肺转移瘤的灵敏度分别达到74.7%和64.0%。Margaritora等^[6]^报道了传统CT和HRCT对诊断肺转移瘤的灵敏度，对诊断 > 1 cm的肺转移瘤，两者均达到了100%的灵敏度，对 < 6 mm的肺转移瘤传统CT和HRCT的灵敏度则分别降至62%和48%，但是总体来说两者对诊断肺转移瘤的灵敏度达到82.1%。另外Fortes等^[7]^报道了PET-CT对诊断肺转移瘤的灵敏度达到了65.7%，对于那些结节直径 > 1 cm的肺转移瘤，灵敏度提升到了87.8%，而对于直径 < 1 cm的肺转移瘤灵敏度仅为29.6%，并且对不同的原发肿瘤引起的肺转移瘤灵敏度也不同，对于肉瘤灵敏度仅为44%，而对鳞状细胞癌则达到93%，因此研究者认为PET-CT不能单独用于对肺转移瘤的术前诊断，但可用于对患者的术前评估。

### 3.2 孤立性肺转移瘤的手术适应证及手术方式

目前已公认的肺转移瘤的手术适应证^[3, 8]^包括：①患者可以耐受本次手术；②术前肺功能测试表明有足够的肺功能储备；③原发病灶已得到切除或有效控制；④无肺外转移灶的证据；⑤除此之外没有更有效的治疗措施。在本次研究中有21例患者同时发现原发肿瘤和肺部转移灶，在原发肿瘤切除后才实施肺转移瘤切除术，手术取得了满意的疗效。其余患者在上述手术适应证下进行手术，也取得了满意的疗效。手术方式的选择需要遵循最大程度的切除肿瘤，最大限度的保留正常肺组织这一基本原则。术式可包括肺楔形切除术、肺段切除术、不规则肺部分切除术以及肺叶切除术和全肺切除术。在完全切除肿瘤的前提下要尽可能减少对患者的创伤。目前已有学者采用VATS切除肺转移瘤，但是以往研究认为^[9, 10]^VATS应该只用于对肺转移瘤的诊断而非治疗，因为在VATS中不能用手对肺进行充分触诊，往往仅能发现胸膜下的病灶，易遗漏自肺表面不能看见或术前影像学检查未能发现的病变，从而造成转移瘤切除不完全。但是近年来随着HRCT及PET-CT在临床上的逐渐普及，对诊断肺转移瘤的灵敏度也在逐渐提高^[5, 6]^，这促进了VATS在治疗肺转移瘤方面的应用。Mutsaerts等^[11]^研究发现采用VATS或常规开胸术治疗肺转移瘤，两种方式的患者术后生存期没有明显不同。Carballo等^[12]^通过研究采用VATS或开胸术治疗肺转移瘤患者的临床资料后发现，VATS组的5年生存率明显高于开胸组(69.6% *vs* 58.8%)。在本次研究中我们使用VATS或者开胸手术切除肺转移瘤，两组5年生存率差别无统计学意义。考虑到VATS具有对患者创伤小、术后恢复快等优点，我们认为对符合手术适应证的患者可以采用VATS行转移瘤切除术，如果术中出现病灶定位困难，可以辅助小切口，以明确定位，并探查全肺有无术前检查遗漏的病灶，尽可能做到转移瘤的完全切除。

### 3.3 影响预后的因素

文献^[13, 14]^报道影响肺转移瘤患者术后生存的主要因素有转移瘤是否完全切除、原发肿瘤的病理类型和转移瘤的数目。在本次研究中有无淋巴结转移和肺叶切除方式是影响患者术后生存的独立因素。本组共有113例患者行肺门及纵隔淋巴结的系统性清扫，其中21例术后病理证实淋巴结转移。无淋巴结转移患者和有淋巴结转移患者5年生存率分别为37.3%和12.5%。Pfannschmidt等^[15]^分析了245例行肺转移瘤切除伴肺门及纵隔淋巴结清扫的患者的临床资料，他们发现18%的患者会出现肺和肺门淋巴结的转移，9%的患者会出现肺、肺门和纵隔淋巴结的转移，5%的患者出现纵隔淋巴结的转移。无淋巴结转移患者的中位生存时间为64个月，出现肺门淋巴结转移的为33个月，而出现纵隔淋巴结转移的只有21个月。因此我们认为在术中应行肺门和纵隔淋巴结的系统性清扫，以便评估患者的预后并指导下一步的治疗。另外，在本次研究中行肺叶切除术患者的预后较好，5年生存率为38.5%。在原发肿瘤中以头颈部癌、结直肠癌和软组织肉瘤的预后较好，5年生存率分别为41.2%、37.8%和36.9%。Petersen等^[16]^报道黑色素瘤患者的预后较差，研究者对1, 720例黑色素瘤出现肺转移的患者的临床资料分析后发现5年生存率仅为13%。头颈部肿瘤患者的预后相对较好，5年生存率可达到20.9%-59%，而年龄、性别、肿瘤大小与患者的预后无关^[4]^。

总之，本次研究通过回顾性分析孤立性肺转移瘤患者的临床资料，发现对诊断明确、符合手术指征的患者行孤立性肺转移瘤切除术，可取得满意的手术疗效。但是目前仍缺乏关于孤立性肺转移瘤的前瞻性随机对照研究，因此对于那些接受手术治疗但效果不佳的患者而言，是否能真正从手术中获益并不明确。因此对于手术治疗孤立性肺转移瘤的疗效有待进一步观察，并需要大规模的临床随机对照研究来进行验证，从而进一步提高对孤立性肺转移瘤的诊断和治疗水平，提高患者的生存率。
